# Membrane fluxes, bypass flows, and sodium stress in rice: the influence of silicon

**DOI:** 10.1093/jxb/erx460

**Published:** 2018-01-11

**Authors:** Rubens Flam-Shepherd, Wayne Q Huynh, Devrim Coskun, Ahmed M Hamam, Dev T Britto, Herbert J Kronzucker

**Affiliations:** 1 Department of Biological Sciences and Canadian Centre for World Hunger Research (CCWHR), University of Toronto, Toronto, Canada; 2 Département de Phytologie, Faculté des Sciences de l’Agriculture et de l’Alimentation (FSAA), Université Laval, Québec, Canada; 3 School of BioSciences, The University of Melbourne, Parkville, Victoria, Australia

**Keywords:** Apoplast, efflux, influx, *Oryza sativa* L, cvs Pokkali and IR29, rice, salt stress, silicon, sodium accumulation, sodium transport, transpirational bypass flow

## Abstract

Provision of silicon (Si) to roots of rice (*Oryza sativa* L.) can alleviate salt stress by blocking apoplastic, transpirational bypass flow of Na^+^ from root to shoot. However, little is known about how Si affects Na^+^ fluxes across cell membranes. Here, we measured radiotracer fluxes of ^24^Na^+^, plasma membrane depolarization, tissue ion accumulation, and transpirational bypass flow, to examine the influence of Si on Na^+^ transport patterns in hydroponically grown, salt-sensitive (cv. IR29) and salt-tolerant (cv. Pokkali) rice. Si increased growth and lowered [Na^+^] in shoots of both cultivars, with minor effects in roots; neither root nor shoot [K^+^] were affected. In IR29, Si lowered shoot [Na^+^] via a large reduction in bypass flow, while in Pokkali, where bypass flow was small and not affected by Si, this was achieved mainly via a growth dilution of shoot Na^+^. Si had no effect on unidirectional ^24^Na^+^ fluxes (influx and efflux), or on Na^+^-stimulated plasma-membrane depolarization, in either IR29 or Pokkali. We conclude that, while Si can reduce Na^+^ translocation via bypass flow in some (but not all) rice cultivars, it does not affect unidirectional Na^+^ transport or Na^+^ cycling in roots, either across root cell membranes or within the bulk root apoplast.

## Teaser

Silicon reduces rice shoot sodium accumulation by blocking transpirational bypass flow or increasing shoot growth, but does not affect transmembrane Na^+^ fluxes or the rapid Na^+^ influx–efflux cycle in roots.

## Introduction

Silicon and sodium are among the most abundant elements in the earth’s crust (Wedepohl, 1995). Neither is essential for the growth of most plants, but both can be beneficial under certain conditions (Epstein, 1999; Kronzucker *et al.*, 2013). In most plants (excepting halophytes), the benefits of Na^+^ provision are generally seen only at low external Na^+^ concentrations ([Na^+^]_ext_), while at higher concentrations, Na^+^ can severely impair growth (Kronzucker *et al.*, 2013). As salinity affects up to a third of all agricultural land globally, this impairment leads to an annual loss in crop productivity amounting to some US$12–27 billion (Ghassemi *et al.*, 1995; Qadir *et al.*, 2014; Munns and Gilliham, 2015). In contrast, reports of Si toxicity in plants are almost non-existent (e.g. Sivanesan *et al.*, 2013), while the beneficial effects of Si on plants have been documented for at least two centuries (Davy, 1815; Epstein, 1999). These benefits often take the form of increased biotic and abiotic stress resistances, but include growth enhancements even under benign conditions (Richmond and Sussman, 2003; Ma, 2004; Fauteux *et al.*, 2005; Epstein, 2009; Coskun *et al.*, 2016*a*). While the mechanisms by which Si exerts such effects remain the subject of much exploration, it is clear that the structural properties of polymerized silicates in plant tissues can produce stems and leaves that are stronger and more erect, and therefore better able to, for instance, resist pathogen and herbivore attack, or intercept photosynthetically active light, compared with plants grown without Si (Liang *et al.*, 2015; Meharg and Meharg, 2015). In addition, Si provision can trigger changes in gene expression (Van Bockhaven *et al.*, 2013; Che *et al.*, 2016; Debona *et al.*, 2017; Hinrichs *et al.*, 2017), but evidence for consequent changes on plant metabolism is scarce at this time.

Globally, rice (*Orzya sativa* L.) provides more calories to sustain the human population than any other crop species (Awika, 2011). It is also a species known to accumulate large amounts of Si in its tissues (Savant *et al.*, 1996; Ma and Takahashi, 2002), and to benefit greatly from Si provision, particularly when challenged with salinity (Matoh *et al.*, 1986; Yeo *et al.*, 1999; Gong *et al.*, 2006). However, rice is also quite susceptible to Na^+^ stress and toxicity (Kronzucker *et al.*, 2013; Muthayya *et al.*, 2014), due to its innate sensitivity to salt, and to its widespread cultivation near marine environments (e.g. the Mekong and Ganges deltas), and in irrigated paddies, which are more prone to salinization than non-irrigated fields.

In rice, the protective properties of Si under salinity have been partly attributed to silicon’s inhibition of a ‘transpirational bypass flow’ of water and solutes from root to shoot (Yeo *et al.*, 1999). Bypass flow is a heritable trait, associated with leaf Na^+^ accumulation and salt sensitivity among rice cultivars (Yadav *et al.*, 1996; Krishnamurthy *et al.*, 2009; Faiyue *et al.*, 2012), and is thought to occur purely via apoplastic (extracellular) pathways, bypassing the filtration barrier of the root endodermis that normally prevents the unrestricted flow of Na^+^ and other solutes into the transpiration stream (Yeo *et al.*, 1999; Gong *et al.*, 2006). Si in the nutrient solution can block bypass routes and thereby greatly reduce the amount of Na^+^ appearing in the shoot via bypass flow in some rice cultivars, an effect that is correlated with improvements in growth under saline conditions (Yeo *et al.*, 1999; Gong *et al.*, 2006). The means by which this blockage occurs is not well understood, but it is probably due to silicate polymerization in the root endodermis and exodermis, particularly in young roots and regions of lateral root emergence (Lux *et al.*, 1999; Ranathunge *et al.*, 2005; Gong *et al.*, 2006; Krishnamurthy *et al.*, 2009; Maathuis *et al.*, 2014). It has also been linked to the promotion of suberization, lignification, and Casparian band formation by Si, in roots of rice and other species (Fleck *et al.*, 2011; Hinrichs *et al.*, 2017).

Despite decades of study, many questions persist about the membrane transport systems responsible for the initial, ‘primary’ acquisition step of Na^+^ by root cells from a saline (or, more specifically, a high [Na^+^]_ext_) environment. These include questions about the molecular identities, functional characteristics, deployment, and integration of the low-affinity transporters that catalyze unidirectional Na^+^ influx and efflux across the plasma membrane (Kronzucker and Britto, 2011; Cheeseman, 2013; Britto and Kronzucker, 2015; Deinlein *et al.*, 2015; Nieves-Cordones *et al.*, 2016). Indeed, our current picture of low-affinity Na^+^ transport at the level of molecular genetics is much less well resolved than that of, for example, high-affinity K^+^ transport (Nieves-Cordones *et al.*, 2016). This might be because Na^+^ is not an essential nutrient for most plants, so selection pressures to evolve or retain Na^+^-specific primary acquisition systems might therefore be weaker. However, evidence from electrophysiological, radiotracer, fluorescence, and other methods has indicated that membrane proteins of various classes may catalyze this critical first step of transport, including such non-selective cation channels (NSCCs) as cyclic nucleotide-gated channels (CNGCs) and glutamate-like receptors (GLRs), a low-affinity cation channel (LCT1), K^+^-selective channels of the Shaker family (such as AKT1), and members of the HKT family of Na^+^ and K^+^ transporters (for details, many recent reviews are available, including Zhang *et al.*, 2010; Kronzucker and Britto, 2011; Maathuis *et al.*, 2014). Interestingly, the ‘secondary’ transporters that help shuttle Na^+^ internally within the plant, once it has been taken up by root cells, are relatively well understood. These include proton-linked antiporters that can move Na^+^ from the cytosol to other compartments, such as the external medium (SOS1), the vacuole (NHX proteins), and the xylem stream (SOS1 and CHX), in addition to HKT proteins that can retrieve Na^+^ from the xylem stream in rice, wheat, and Arabidopsis (Kronzucker and Britto, 2011; Deinlein *et al.*, 2015). Given that Si-mediated salt tolerance could conceivably proceed via interactions between Si and any of the above transporters, it is perhaps surprising that this topic has been virtually unexplored (Coskun *et al.*, 2016*a*).

Another means by which Si could potentially exert control over Na^+^ fluxes within the root system is by altering the transport properties of the root apoplast, a possibility that calls for a brief background discussion. We have previously shown (Britto and Kronzucker, 2006) that, as the external concentration of an ion rises in the low-affinity range, the ratio of unidirectional efflux to influx for that ion, namely out of and into a root system, also tends to rise, and approach unity (as measured using radiotracers). At the same time, the magnitude of influx tends to rise linearly in this range, resulting in an increased, and more intensely cyclical, movement of ions into and out of the root. For Na^+^, such rapid ion cycling has been reported in many species, including *Arabidopsis thaliana*, rice, barley, wheat, and *Puccinellia tenuiflora* (Essah *et al.*, 2003; Malagoli *et al.*, 2008; Britto and Kronzucker, 2009; Wang *et al.*, 2009), as might be expected, given that Na^+^ is of particular interest when present at high (saline) concentrations. Rapid Na^+^ cycling often entails efflux:influx ratios of ≥0.9 (Maathuis *et al.*, 2014; Britto and Kronzucker, 2015), and unidirectional fluxes that are orders of magnitude larger than the net flux needed to accumulate Na^+^ to concentrations found in the plant; they can also be orders of magnitude larger than what is typically measured for nutrient ions crossing the plasma membrane under high-affinity conditions (Britto and Kronzucker, 2009, 2015; Maathuis *et al.*, 2014). Rapid Na^+^ cycling is commonly assumed to proceed across the plasma membrane of root cells (e.g. Malagoli *et al.*, 2008; Maathuis *et al.*, 2014); therefore, we have termed this description of Na^+^ transport the ‘rapid transmembrane sodium cycling’ (RTSC) model (Britto and Kronzucker, 2015). However, several lines of evidence suggest that a significant fraction of rapidly cycling Na^+^ does not cross membranes at all, but may instead cycle extracellularly, into and out of the root cortical apoplast, driven by diffusion and the mass flow of water into roots. These lines of evidence include the following five: (i) the unrealistically large amounts of metabolic energy that such large fluxes would require were they predominantly trans-membrane, exceeding in some cases the limits of what can be provided by cellular respiration (Malagoli *et al.*, 2008; Britto and Kronzucker, 2009); (ii) the unrealistically high cytosolic Na^+^ concentrations that would be derived from compartmental analyses of these fluxes, assuming they were trans-membrane (Kronzucker *et al.*, 2013); (iii) the large fractions of unidirectional Na^+^ tracer fluxes that are resistant to powerful inhibitors, especially in the case of Na^+^ efflux, which is an energy-demanding process according to the RTSC model (Malagoli *et al.*, 2008; Britto and Kronzucker, 2009, 2015; Kronzucker and Britto, 2011); (iv) the striking similarities in tracer release (efflux) kinetics between the fluorescent apoplastic tracer PTS (trisodium 8-hydroxypyrene-1,3,6-trisulfonic acid), and both Na^+^ and K^+^ radiotracers in the high concentration range (Yeo *et al.*, 1987; Anil *et al.*, 2005; Coskun *et al.*, 2016*b*); and (v) the recent detailed demonstrations of rapid apoplastic cycling of K^+^ in barley and Arabidopsis roots at high external concentrations (Coskun *et al.*, 2016*b*). Experimental support for the RTSC model, on the other hand, has been hampered by our limited mechanistic understanding of unidirectional, low-affinity Na^+^ fluxes across root plasma membranes (see above), including that of Na^+^ efflux from root cells to the external medium (Britto and Kronzucker, 2015). Only one transporter (SOS1) has been found that could potentially catalyze this critical step, but its participation in large-scale root Na^+^ cycling is doubtful because its expression is limited mainly to the root tip and the xylem parenchyma (Hamam *et al.*, 2016); moreover, no differences in kinetic patterns of Na^+^ tracer release were found between the wild type and *sos1* mutants of Arabidopsis (Ding and Zhu, 1997; Shi *et al.*, 2002; Maathuis *et al.*, 2014; Britto and Kronzucker, 2015). Very little is known about the Si influence on a putative apoplastic cycling of Na^+^ within the plant root (Malagoli *et al.*, 2008), but it is worthwhile addressing this possibility, given the known reduction by Si of apoplastic, transpirational bypass flow in rice (see above).

In the present study, we examined the influence of Si on growth, root to shoot translocation of Na^+^, and the unidirectional fluxes that cycle Na^+^ through the root, in hydroponically grown, salt-tolerant and salt-sensitive rice (Pokkali and IR29, respectively).

## Materials and methods

### Plant material and growth conditions

Rice (*Oryza sativa* L.) cultivars were chosen due to their contrasting salinity tolerance; IR29 is ‘salt sensitive’ and Pokkali is ‘salt tolerant’ (Gregorio *et al.*, 1997; see the Results and Discussion). Seed material was provided by the International Rice Research Institute (Los Banos, Philippines). Seeds were surface-sterilized with 1% (v/v) sodium hypochlorite for 15 min, rinsed for 3 h with dH_2_O, germinated in aerated dH_2_O for 48 h, and floated on aerated, modified Johnson’s solution [2 mM MgSO_4_, 0.5 mM K_2_SO_4_, 0.5 mM (NH_4_)_2_SO_4_, 0.3 mM KH_2_PO_4_, 0.3 mM CaCl_2_, 0.1 mM Fe-EDTA, 20 µM H_3_BO_3_, 9 µM MnCl_2_, 1.5 µM CuSO_4_, 1.5 µM ZnSO_4_, 0.5 µM Na_2_MoO_4_]. Si was supplied as sodium silicate (Sigma-Aldrich; 26.5% SiO_2_, 10.6% Na_2_O), added in sufficient quantity to produce a final Si concentration of 1.67 mM (or, in the case of Fig. 5, 3.0 mM). Examination of a 0 mM [Na^+^]_ext_ condition was not possible due to the form of Si (sodium silicate) used in this study. Use of a dual-channel flame photometer (Model 2655-10; Cole-Parmer, Anjou, QC, Canada) confirmed the manufacturer’s specifications of sodium content, and 1.32 mM NaOH was supplied to (–Si) control treatments to account for the exogenous Na^+^ in +Si treatments. This allowed approximately equal amounts of H_2_SO_4_ to adjust both control and Si-supplemented nutrient solutions to a pH of 6.30–6.35. Na^+^ concentrations above these background amounts were provided as NaCl, in amounts as indicated.

To ensure that plants were maintained at a nutritional steady-state, solutions were completely exchanged on days 9, 13, 17, and 20. Si and NaCl treatments were imposed on day 2 (post-sterilization), and continued for 19 d in total, with plants sampled on day 21. Plants were cultivated in the same controlled growth chamber in which experiments were later conducted, having a 12 h:12 h, light:dark cycle, with cool-white fluorescent tubes (F96T12/CWW/VHO; Sylvania, Danvers, MA, USA) providing an irradiation of 425 µmol photons m^−2^ s^−1^ (Silhouette High Output F54T5/850HO; Philips Electronics Ltd, Markham, ON, Canada), a relative humidity of 70%, and temperatures of 30 °C and 20 °C in the daytime and night-time, respectively. During the growth period, growth solutions were regularly checked via flame photometry to ensure that nutrient depletion was <15% (using K^+^ as a proxy).

### Tissue fresh weight, K^+^ content, and Na^+^ content

Measurement of tissue FW, DW, and K^+^ and Na^+^ content was performed as described previously (Britto *et al.*, 2010; Schulze *et al.*, 2012), on plants grown at [Na^+^]_ext_ of 5, 10, 25, and 50 mM. Briefly, roots of intact 21-day-old seedlings were placed in 10 mM CaSO_4_ for 5 min to release extracellular cations. Roots were then detached from shoots and spun in a low speed centrifuge for 30 s to remove surface water. FW and DW of the tissue samples were determined before and after, respectively, oven-drying for 3 d at 85–90 °C. Tissue samples were then pulverized and digested for an additional 3 d in 30% (v/v) HNO_3_. K^+^ and Na^+^ concentrations of the tissue digests were measured with a dual-channel flame photometer (see above).

### Determination of cultivar- and condition-specific EC_50_

**Fig. 1.**
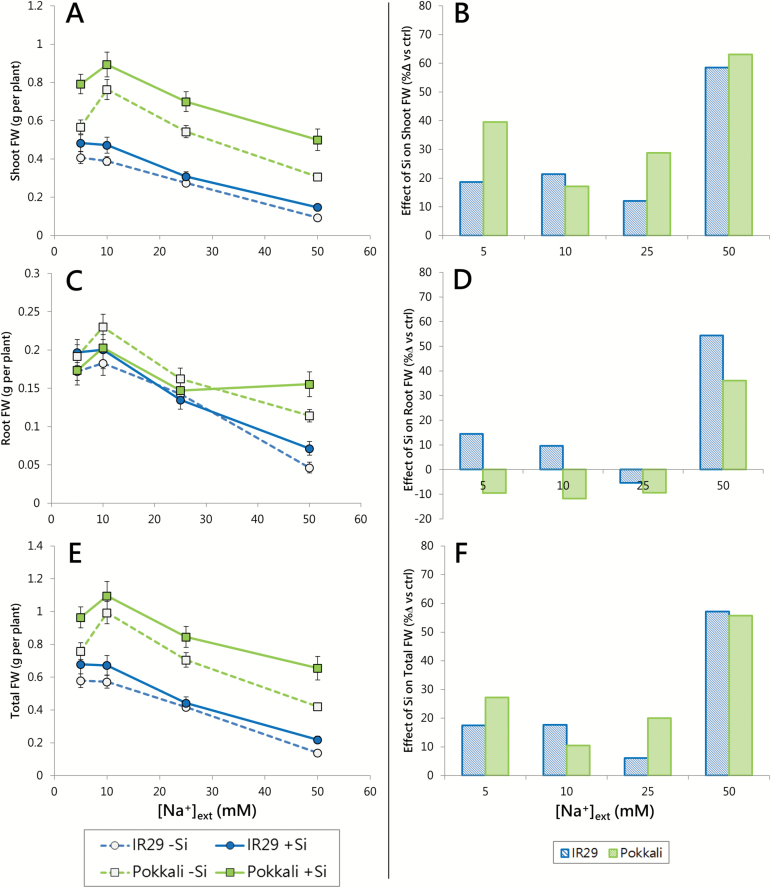
Fresh weight of 21-day-old seedlings of IR29 (circles) and Pokkali (squares), in response to NaCl (5–50 mM) and Si (0 or 1.67 mM) treatments, imposed when seedlings were 2 d old. (A, C, E) Shoot, root, and total FW. (B, D, F) Percentage increases in shoot, root, and total FW in Si-treated plants, relative to controls. FW data are represented as the mean ±SEM (*n*=11–12). Dotted lines and open symbols, 0 mM Si; solid lines and filled symbols, 1.67 mM Si. Measurements taken at 30 °C in seedlings grown under a 30/20 °C (day/night) temperature cycle. Statistical analysis of these data can be found in Supplementary Table S1, and corresponding dry weight data in Supplementary Fig. S1.

Linear regression of shoot FW data measured at 10–50 mM [Na^+^]_ext_ revealed the effective concentration at which [Na^+^]_ext_ exerted a 50% suppression on shoot FW (EC_50_) for each condition. This was determined relative to shoot FW at 10 mM [Na^+^]_ext_, because, at this [Na^+^]_ext_, Pokkali exhibited maximal shoot FW, while IR29 experienced no deleterious effects (see the Results). In addition, shoot FW decreased linearly (*r*^2^=0.98–0.99) between 10 mM and 50 mM [Na^+^]_ext_ (see Fig. 1A). Condition-specific EC_50_ values were found to be 36.2 mM and 37.6 mM [Na^+^]_ext_ for IR29 in 0 mM and 1.67 mM Si, respectively, and 42.3 mM and 54.3 mM [Na^+^]_ext_ for Pokkali in 0 mM and 1.67 mM Si, respectively. All PTS, radiotracer, and electrophysiological experiments were conducted in plants grown and measured at the approximate EC_50_ values of 35 mM [Na^+^]_ext_ for IR29, and 50 mM [Na^+^]_ext_ for Pokkali, except for experiments examining changes in the plasma membrane electrical potential differences (∆∆Ψ; Fig. 4), which were conducted in plants grown at 5 mM [Na^+^]_ext_, and in response to increasing [Na^+^]_ext_, and unidirectional influx experiments (Fig. 5; these were conducted at 25 mM [Na^+^]_ext_, prior to the determination of EC_50_ values).

### Transpirational bypass flow

Transpirational bypass flow from root to shoot via the apoplast was measured using PTS, an established apoplastic tracer (Moon *et al.*, 1986; Yeo *et al.*, 1987, 1999; Krishnamurthy *et al.*, 2011), in plants grown at their cultivar-specific EC_50_ (i.e. 35 mM and 50 mM [Na^+^]_ext_ for IR29 and Pokkali, respectively). Roots of intact 21-day-old seedlings were immersed for 24 h (12 h light and 12 h dark) in growth solution that contained 0.01% (w/v) PTS (190 µM). Following this, roots were rinsed twice in PTS-free growth solution, first for 5 s and then for 5 min. Plant shoots and roots were then harvested, weighed, pulverized with a mortar and pestle under liquid N_2_, and extracted for 2 h in dH_2_O. On average, the pH of the extracts was 5.75, at which a single fluorescence excitation peak (at 403 nm) is expected for PTS (Faiyue *et al.*, 2010). Therefore, we measured PTS fluorescence at λ_excitation_=403 nm and λ_emission_=510 nm (λ_emission_ is relatively pH insensitive; Faiyue *et al.*, 2010) using a fluorescence spectrophotometer (Synergy 4 Hybrid Microplate Reader; BioTek, Winooski, VT, USA), and expressed it as fluorescence relative to the loading vessel, per gram of shoot FW. Transpiration was measured gravimetrically. Calculations of bypass flow were made using tissue PTS concentrations and transpiration volumes, according to established procedures (e.g. Garcia *et al.*, 1997; Anil *et al.*, 2005).

### Electrophysiology

Steady-state (resting) electrical potential differences (∆Ψ) across the plasma membrane of epidermal and cortical root cells, situated 1–3 cm from the root tip, were measured as described previously (Schulze *et al.*, 2012). Briefly, at an ambient temperature of 25 °C, roots of intact 21-day-old seedlings (grown at their cultivar-specific EC_50_ values) were immersed in growth solution within a plexiglass cuvette (125 ml volume) mounted on a light microscope (Leica DME; Leica Microsystems Inc., Concord, ON, Canada). Root cells were impaled with borosilicate glass microelectrodes that were back-filled with 3 M KCl, and ∆Ψ values were recorded using an electrometer (Duo 773; World Precision Instruments Inc., Sarasota, FL, USA). ∆Ψ values clustered into two distinct populations (see Nieves-Cordones *et al.*, 2008); the more negative set of values was considered to represent potentials across the plasma membrane (i.e. the electrode tip was in the cytosol), as they closely resembled typical plasma membrane electrical potential values from other work, but not the much less polarized values found across the tonoplast (Walker *et al.*, 1996; Carden *et al.*, 2003; Nieves-Cordones *et al.*, 2008).

To observe changes in ∆Ψ (i.e. ∆∆Ψ) in response to increases in [Na^+^]_ext_, plants were grown on a background of 5 mM [Na^+^]_ext_ and impaled as described above. After the membrane potential was recorded, peristaltic pumps were switched on to exchange nutrient solution in the cuvette at a rate of ~7.5 ml min^−1^, and to increase [Na^+^]_ext_ to 10 mM. Once a new stable membrane potential was reached (within 60–100 s), this process was repeated twice, with further step-ups to 50 mM and 100 mM [Na^+^]_ext_.

### General preparations for work with ^24^Na^+^ radiotracer

Two days prior to experiments (19 d after seeds were surface-sterilized), seedlings were bundled together in groups of 4–5 at the basal shoot by a 0.5 cm high plastic collar. Fluxes of Na^+^ were determined using ^24^Na^+^ (*t*_1/2_=14.96 h; as ^24^NaCl, provided by the McMaster Nuclear Reactor, Hamilton, ON, Canada), as described in detail elsewhere (Siddiqi *et al.*, 1991; Malagoli *et al.*, 2008; Coskun *et al.*, 2014). Briefly, roots of bundled, 21-day-old seedlings were immersed for various load times (see below) in radioactive loading solutions identical to growth solutions, except that they contained ^24^Na^+^ in addition to non-radioactive Na^+^. Na^+^ concentrations were equal to the EC_50_ values for each cultivar (usually; see above). After loading, bundles were removed from radioactive solution and returned to non-radioactive growth solution for various desorption times. Roots were separated from shoots and spun for 30 s in a low-speed clinical centrifuge (to remove surface film) before weighing. Measurements of radioactivity (counts per minute; cpm) in eluates, roots, and shoots were made using a gamma counter (Cobra Quantum 5003; PerkinElmer Inc., Waltham, MA, USA) equipped with a 3 inch sodium iodide scintillation crystal that detected γ rays emitted from excited ^24^Mg, the daughter nuclide produced when ^24^Na^+^ undergoes β^−^ decay. Counts were automatically corrected for radioactive decay. Where appropriate, cpm values were converted to molar quantities of Na^+^, using the specific activities of the loading solutions. All experiments were done during the light period, with the exception of those utilizing a 24 h load, which were done with 12 h light and 12 h dark. Additional details for particular flux protocols are described below.

### Unidirectional Na^+^ influx

Roots of intact seedlings were loaded for 2 min in radioactive solution, and then desorbed in two sequential steps (first for 5 s and then for 7 min) in non-radioactive solution. Plants were then harvested and measured for radioactivity as described above.

### Shoot ^24^Na^+^ content

Roots of intact seedlings were immersed for 24 h in aerated, radioactive nutrient solution (as above), then desorbed in two sequential steps (for 5 s and then 7 min) in non-radioactive growth solution prior to harvesting and counting.

### Na^+^ efflux

Roots of intact seedlings were immersed for 1 h in growth solution supplemented with ^24^Na^+^, before seedlings were removed and secured to the inside of glass efflux funnels (Coskun *et al.*, 2014). Roots were then eluted of radioactivity with successive 13 ml aliquots of non-radioactive growth solution every 1.5 min, for a total of 39 min of elution. A subset of efflux experiments was conducted with the final 12 eluates (from the 22.5 min point onwards in the elution protocol) being supplemented with 1 mM KCN and 1 mM salicylhydroxamic acid (SHAM), standard inhibitors of the cytochrome *c* oxidase and alternative oxidase respiratory pathways, respectively. Unidirectional influx and efflux were determined according to the method of Siddiqi *et al.*, 1991.

### Statistics

To determine tissue ion content, replicates consisted of the combined FW of 4–5 plants, with each condition replicated 11–12 times over four experiments. For electrophysiology experiments, replicates consisted of a single plant, with each condition replicated 3–9 times. For Na^+^ influx, efflux, and PTS florescence measurements, replicates consisted of a bundle of 4–5 plants attached together at the shoot base by a collar made from plastic tubing, with each condition replicated 3–6 times. Throughout, results are expressed as the mean ±SEM. Means were compared using two-way ANOVA (Supplementary Tables S1, S3 at *JXB* online) or Student’s *t*-tests with Bonferroni post-hoc corrections (Supplementary Tables S2, S4) (*P*<0.05), and slopes were compared using analysis of covariance (ANCOVA; *P*<0.05).

## Results and Discussion

### Plant growth and ion concentrations

**Fig. 2.**
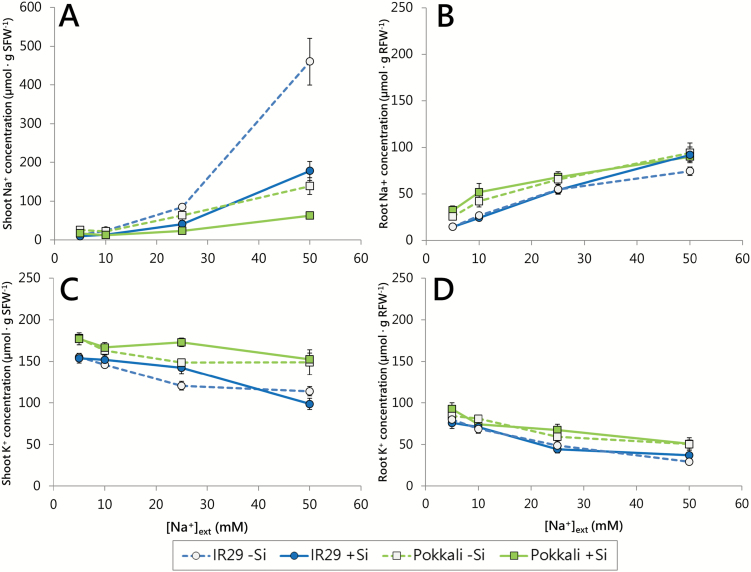
Na^+^ (A, B) and K^+^ (C, D) concentrations in shoots (A, C) and roots (B, D) of 21-day-old seedlings of IR29 (circles) and Pokkali (squares), in response to NaCl (5–50 mM) and Si (0 or 1.67 mM). Data are normalized per gram of organ FW and are represented as the mean ±SEM (*n*=11–12). Dotted lines and open symbols, 0 mM Si; solid lines and filled symbols, 1.67 mM Si. Measurements taken at 30 °C in seedlings grown under a 30/20 °C (day/night) temperature cycle. For FW:DW ratios, see Supplementary Fig. S3.

At the outset, it was important to confirm in our system the NaCl sensitivities and ion contents of the two rice cultivars (Figs 1, 2). Pokkali, a tall (non-dwarf), variable landrace, is traditionally grown in coastal southern India, and is adapted to marshy, saline environments, including mixed agriculture/aquaculture systems (Yeo *et al.*, 1991; Gregorio *et al.*, 1997; Anil *et al.*, 2005). IR29, in contrast, is a modern semi-dwarf cultivar which, although high yielding, is notably salt sensitive (Gregorio *et al.*, 1997; Peng and Khush, 2003; Razzaque *et al.*, 2017). True to form, Pokkali showed higher shoot and overall growth than IR29 under all conditions (*t*-test, *P*<0.05; Fig. 1A, E; Supplementary Fig. S1; Supplementary Tables S1, S2), demonstrating a cardinal difference between tall and semi-dwarf varieties that can be found even at the early seedling stage (Yeo *et al.*, 1991). Also true to form, Pokkali was substantially more salt resistant than IR29 (Fig. 1). We estimated that the ‘effective concentrations’ (EC_50_) at which NaCl exerted a 50% decline in growth (relative to growth at 10 mM) were 50 mM for Pokkali and 35 mM for IR29 (see the Materials and methods). However, the slopes of declining plant FW with increasing [Na^+^]_ext_ were similar for the two cultivars, but because Pokkali had a much greater peak FW (nearly double that of IR29), its growth suppression was, percentage-wise, not as pronounced. It should be noted that, because osmotic stress is one of the components leading to growth decline under salinity (Munns and Tester, 2008), this decline should be attributed not just to Na^+^ but also at least partially to Cl^−^.

Interestingly, between 5 mM and 10 mM [Na^+^]_ext_, Pokkali showed an increase in shoot FW, of 13 and 35% with and without Si, respectively; modest increases were also seen in roots (Fig. 1). While positive effects of moderate salinity on growth are common in many species, especially halophytes (Subbarao *et al.*, 2003; Kronzucker *et al.*, 2013), they have been scarcely reported in rice (cf. Dionisio-Sese and Tobita, 1998). No NaCl-dependent growth increase was observed with IR29, however, which may be indicative of its salt sensitivity. In the future, it may be worthwhile to explore the question of growth enhancement due to small amounts of NaCl in a larger range of rice cultivars, to ascertain whether this is a general feature of salt tolerance in this species. It may also be worth considering the potential utility of NaCl as a beneficial nutrient for some cultivars, and the mechanisms that underlie its benefits.

Si provision significantly increased shoot FW in both cultivars (most noticeably in Pokkali) at almost all concentrations of Na^+^, with smaller and less consistent effects on root FW (ANOVA, *P*<0.05; Fig. 1; Supplementary Table S1). Although the benefits of Si to plants are generally described in terms of improved stress tolerance (Epstein, 1999; Richmond and Sussman, 2003; Ma, 2004), we found that Si substantially increased growth rates even in the absence of salt stress (e.g. at 5 mM and 10 mM [Na^+^]_ext_). This result agrees with some previous studies (e.g. Gong *et al.*, 2006) but not with others (Matoh *et al.*, 1986; Yeo *et al.*, 1999). In addition, the slopes of the [Na^+^]_ext_-dependent decline in shoot FW was Si independent (ANCOVA, *P*>0.05; Fig. 1A). Nevertheless, when direct percentage-wise comparisons were made between control and Si treatments for a given cultivar at a given [Na^+^]_ext_, it was clear that the beneficial effects of Si on shoot, root, and total FW were maximal at the highest [Na^+^]_ext_ of 50 mM (Fig. 1B, D, F).

In addition to stimulating growth, Si increased the percentage of dry matter in Pokkali under all [Na^+^]_ext_ conditions, and in IR29 at low [Na^+^]_ext_ (5–10 mM; Supplementary Figs S1–S3). A similar Si-stimulated increase was previously seen by Gong *et al.* (2006), in the salt-sensitive, semi-dwarf rice cultivar IR36, although it was not as pronounced as in our study, and was found only in the absence of Na^+^. In their study and ours, percentage dry matter also tended to increase with increasing [Na^+^]_ext_ (Supplementary Fig. S3). Two independent effects appear to be occurring here, that might be explained by two distinct mechanisms: (i) Si stimulates production of dry matter via increases in photosynthesis (Detmann *et al.*, 2012), and by the accumulation of Si itself, which can reach up to 10% of total DW in rice (Ma and Takahashi, 2002); and (ii) Na^+^ increases the relative amount of dry matter via dehydration of tissue (Munns, 2002).

In both cultivars, losses in FW due to increasing [Na^+^]_ext_ were associated with significant increases in tissue [Na^+^] (Fig. 2A, B), and reductions in tissue [K^+^] (Fig. 2C, D) (ANOVA, *P*<0.05; Supplementary Table S3). Pokkali exhibited lower [Na^+^] and higher [K^+^] in the shoots compared with IR29 under all conditions (Fig. 2C), and also generally maintained slightly higher levels of both K^+^ and Na^+^ in the root (Fig. 2B, D). These results are consistent with many studies that have shown an inverse correlation between plant growth and Na^+^ accumulation (and K^+^ loss) in leaves under saline conditions (Munns, 2002; Tester and Davenport, 2003; Chen *et al.*, 2007; Møller *et al.*, 2009; Coskun *et al.*, 2013). Although there are many exceptions to this tendency, such as in wheat (e.g. Genc *et al.*, 2007), barley (Mian *et al.*, 2011), and Arabidopsis (Rus *et al.*, 2004), it appears to be quite reliable in rice (Matoh *et al.*, 1986; Garcia *et al.*, 1995; Gong *et al.*, 2006; Coskun *et al.*, 2013; cf. Yeo *et al.*, 1990).

Si strongly decreased shoot [Na^+^] in both Pokkali and IR29 (ANOVA, *P*<0.05; Fig. 2A; Supplementary Table S3), but no consistent effect of Si on root [Na^+^], or either root or shoot [K^+^], was seen in either cultivar (Fig. 2B–D; Supplementary Table S3). In the shoots of both IR29 and Pokkali, this resulted in a pronounced drop in the [Na^+^]:[K^+^] ratio due to Si application (Supplementary Table S4). Our results, and similar results reported by Gong *et al.* (2006), are consistent with the finding that a lower shoot [Na^+^]:[K^+^] ratio can predict lower yield losses in rice crops growing under salinity (Asch *et al.*, 2000). However, at the highest [Na^+^]_ext_ of 50 mM, the relationship between this ratio and plant growth was much more pronounced in Pokkali than in IR29. At this concentration, Si decreased the shoot [Na^+^]:[K^+^] ratio in Pokkali by 0.4 (Supplementary Table S4), and increased the total FW by 56% (0.23 g per plant; Fig. 1E). In contrast, in Si-treated IR29, the much larger decrease (of 1.5) in shoot [Na^+^]:[K^+^] was associated with a nearly identical FW gain (57%, or 0.08 g per plant). This disparity brings to mind the work of Yeo *et al.* (1990), who, in a study of some 150 genotypes of rice, concluded that shoot [Na^+^] accounted only for a small proportion of the total variability of survival under salinity.

### Sodium transport from root to shoot

The movement of solutes from root to shoot generally involves a series of trans-membrane transport steps in the root, which allow solutes to cross the hydrophobic barriers of the root endodermis, and thus enter the xylem stream. In rice, however, a large fraction (as much as 50%; Maathuis *et al.*, 2014) of Na^+^ translocated from root to shoot occurs via an apoplastic bypass of endodermal barriers and membrane transport (Yeo *et al.*, 1987; Ochiai and Matoh, 2002; see the Introduction). In transpirational bypass flow, ions may enter the xylem without ever crossing a cell membrane, through gaps where the endodermis is undeveloped (as in younger parts of the root), or where lateral roots emerge (Ranathunge *et al.*, 2005). Although many questions remain regarding this model (Faiyue *et al.*, 2010; Krishnamurthy *et al.*, 2011), it is clear that Si can play a critical role in reducing shoot [Na^+^] in rice, blocking bypass flow most probably via the formation of silica gels or polysilicic acids, as well as through the enhancement of suberization and lignification (Yeo *et al.*, 1999; Gong *et al.*, 2006; Shi *et al.*, 2013; Hinrichs *et al.*, 2017).

**Fig. 3.**
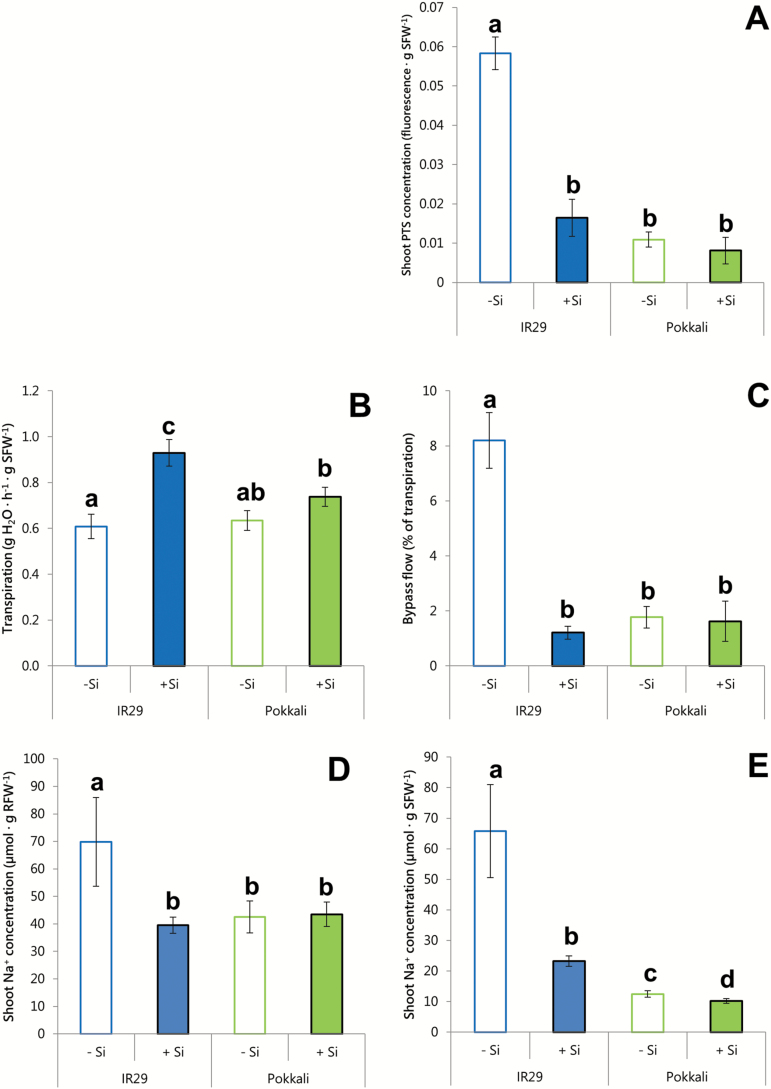
Root to shoot transport in 21-day-old seedlings of IR29 and Pokkali, grown and measured at their cultivar-specific EC_50_ values of 35 mM and 50 mM [Na^+^]_ext_, respectively, and in response to 0 or 1.67 mM Si. (A) Shoot PTS concentration, (B) transpiration, (C) bypass flow, (D) shoot ^24^Na^+^ accumulation normalized to root fresh weight (RFW), and (E) shoot ^24^Na^+^ accumulation normalized to shoot fresh weight (SFW). Plants were exposed to PTS or ^24^Na^+^ for 24 h before desorption and harvesting. PTS fluorescence is expressed relative to the fluorescence of the loading vessel. Different lower case letters (a, b, c, d) indicate significant differences between conditions (*t*-test, *P*<0.05). Data are represented as the mean ±SEM (*n*=3–7). Empty bars with coloured borders, 0 mM Si; Filled bars with black borders, 1.67 mM Si. Measurements taken at 30 °C in seedlings grown under a 30/20 °C (day/night) temperature cycle.

Our estimates of transpirational bypass flow using PTS, a fluorescent compound that does not cross membranes and is therefore often used as an apoplastic tracer for bypass flow (Peterson *et al.*, 1981; Yeo *et al.*, 1987; Anil *et al.*, 2005; Faiyue *et al.* 2012), support the idea of such a role for Si in IR29, but not in Pokkali (Fig. 3). In IR29, Si provision decreased the accumulation of PTS in the shoot by 70%, but had no significant effect in Pokkali, which, regardless of Si treatment, accumulated PTS to nearly the same low level as Si-treated IR29 (Fig. 3A). Because bypass flow is conventionally and most meaningfully expressed as a percentage of transpiration (Yeo *et al.*, 1987), we also measured transpiration (Fig. 3B) and then estimated bypass flow (Fig. 3C) in both cultivars, grown at their respective EC_50_ values. In parallel with the PTS results, Si dramatically reduced bypass flow in IR29 (from 5.6% to 1% of transpiration), but had no effect in Pokkali (Fig. 3C). Moreover, bypass flow in Pokkali was very low (1.2–1.5%) with or without Si, and comparable with that of Si-treated IR29. This suggests that these values represent a ‘constitutive’ or baseline flow that cannot be further reduced by Si application. Indeed, they are only moderately greater than bypass flow values found in wheat (~0.4%), a species for which this pathway of Na^+^ translocation has ‘little relevance’ (Garcia *et al.*, 1997). Our results for Pokkali, however, contrast with those of Anil *et al.* (2005), who reported much greater bypass flow in this cultivar, ranging from 8.9% to 54% (under conditions of 0 mM Ca^2+^ and either 0 mM or 200 mM Na^+^, respectively). We find that these values are somewhat problematic, however, as it is widely held that bypass flow is rarely, if ever, more than a few percent of transpiration as a whole (Yeo *et al.*, 1987, 1999; Garcia *et al.*, 1997). Indeed, our survey of published bypass flow values in rice plants (Supplementary Table S5) indicates that the values reported by Anil *et al.* (2005) are up to two orders of magnitude higher than those found in other work. A possible explanation of the unusually high rates in the study of Anil *et al.* (2005) may lie in the very low transpiration rates reported therein.

It is worth noting that Si increased transpiration in both cultivars (albeit significantly in IR29 only; Fig. 3B). This effect was previously seen in two cultivars of rice (GR4 and IR26) by Yeo *et al.* (1999), who concluded in their study that the reduced flux of Na^+^ to the shoot of Si-treated plants could not have been due to a decrease in transpiration. Interestingly, however, an Si-dependent increase in transpiration would be expected to stimulate bypass flow (Faiyue *et al.*, 2010), while Si deposition along pathways leading to the root vasculature would be expected to block it. These opposing effects of Si may be approximately equal in Pokkali, resulting in no net change in bypass flow with Si, but very unequal in IR29, resulting in a large reduction in the overall flow rate (Fig. 3C). In both cultivars, however, increased transpiration due to Si provision might be beneficial to photosynthetic production, without carrying the penalty of increased transpiration-driven Na^+^ transport to the shoot, via bypass flow.

Consistent with the Si-dependent reduction of bypass flow in IR29, there was a significant Si-dependent reduction in the amount of ^24^Na^+^ (radiotracer) that was translocated from roots to shoots of IR29 over 24 h (at its EC_50_; Fig. 3D, E). This was found whether the tracer flux was normalized to the FW of the roots (RFW; Fig. 3D) or the shoots (SFW; Fig. 3E). These two normalization procedures provide distinct and complementary information about Na^+^ translocation: the first gives the capacity of roots to transfer a quantity of Na^+^ to the shoot, while the second gives the resultant concentration of Na^+^ in the shoots. Results normalized to RFW are, in addition, expected to be indicative of bypass flow of Na^+^, given that bypass flow is initially a root phenomenon, occurring via lateral roots and break points in the root endodermis (and exodermis, where present; see below). In Pokkali, unlike IR29, data normalized in this way showed that Si had no effect on root ^24^Na^+^ translocation capacity (Fig. 3D), which was consistent with the lack of an Si effect on bypass flow in this cultivar (Fig. 3C). On the other hand, when expressed on an SFW basis (Fig. 3E), the data showed that Si did in fact significantly lower the resulting concentration of ^24^Na^+^ translocated to the leaves (relative to the –Si condition); here, the effect was consistent with the Si-dependent reduction in the concentration of the leaf Na^+^ accumulated over the lifetime of the plant (Fig. 2A).

In Pokkali, then, the decline in shoot [Na^+^] due to Si cannot be explained by a decline in bypass flow. However, it can be partially explained by the pronounced stimulation of leaf growth, and shoot to root ratio, we found under Si nutrition in this cultivar, that would result in a dilution of the shoot Na^+^ pool (Greenway and Munns, 1980). However, the reduction in shoot [^24^Na^+^] due to Si, measured after 24 h of tracer application, was only ~20% (Fig. 3C), while over the lifetime (3 weeks) of the plant the reduction in [Na^+^]_leaf_ due to Si was ~50% (Fig. 2A), suggesting that other [Na^+^]-reducing mechanisms might be at play in this cultivar. One possibility is that, over the longer term, an Si-stimulated shoot to root recirculation of Na^+^ takes place in Pokkali, contributing to a lowered concentration in leaves. However, Maathuis *et al.* (2014) estimated that Na^+^ recirculation can potentially reduce shoot [Na^+^] only by ~5–7%, so it is probably not a major contributor to the much larger overall reduction in Pokkali. Another factor that should be considered here is that Si reduction of leaf Na^+^ accrual in Figs 2A and 3C will be affected by the very different time scales involved in the two experimental situations.

### Sodium cycling in roots, and transport across root cell membranes

It was of fundamental importance to examine the influence of Si not only on the translocation of Na^+^ from root to shoot in the two cultivars, but also on the influx of Na^+^ into roots from the external medium, and the efflux of Na^+^ from roots back into the medium. Under salinity, these two opposing, and nearly equal, fluxes maintain a rapid cycling of Na^+^ through the roots of many plant species, and represent, by far, the largest reported Na^+^ fluxes known in plants (see the Introduction). However, to our knowledge, no previous examination of the role Si might play in either root Na^+^ cycling or membrane transport of Na^+^, has been conducted, apart from a modest analysis of ^24^Na^+^ efflux from IR29 roots (Malagoli *et al.*, 2008). In that study, it was found that Si provision did not alter efflux traces in IR29, grown and measured at 25 mM [Na^+^]_ext_.

**Fig. 4.**
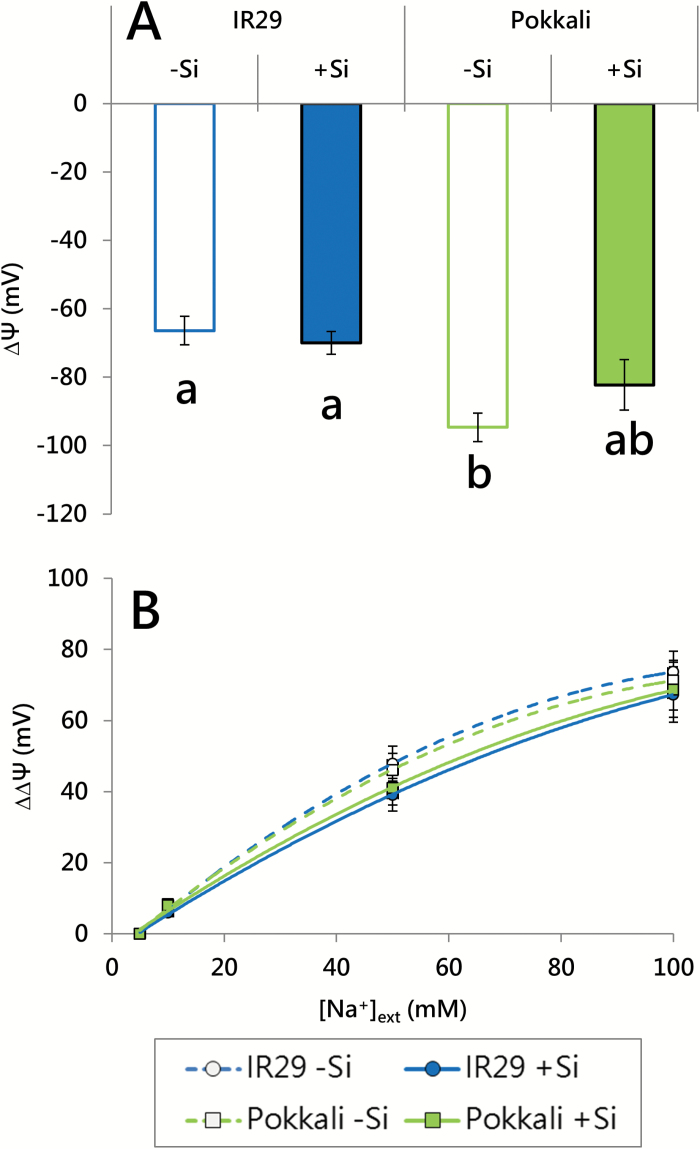
Electrical potential differences (∆Ψ) across the plasma membrane under steady-state conditions (A) and in response to changes in [Na^+^]_ext_ (∆∆Ψ; B), in 21-day-old seedlings of IR29 and Pokkali, grown with or without Si (1.67 mM). Measurements at steady-state conditions occurred at cultivar-specific Na^+^ EC_50_ values (35 mM and 50 mM [Na^+^]_ext_ for IR29 and Pokkali, respectively). Measurements of membrane potential differences in response to changing [Na^+^]_ext_ began at an initial [Na^+^]_ext_ of 5 mM, at which plants had been reared (B). Different lower case letters (a, b) indicate significant differences between conditions (A; *t*-test, *P*<0.05). Data are represented as the mean ±SEM (*n*=5–7). Measurements taken at 30 °C in seedlings grown under a 30/20 °C (day/night) temperature cycle.

Here, we found that Si had no effect on steady-state electrical potential differences (∆Ψ) across the plasma membranes of root cells in either cultivar (Fig. 4A). This indicates that Si did not alter at least one major component of the thermodynamic gradient that potentially drives Na^+^ into root cells (the other being the [Na^+^] gradient across the plasma membrane). Patterns of membrane depolarization resulting from stepwise increases of external [Na^+^], which are indicative of electrogenic (possibly channel-mediated) Na^+^ permeation into cells (Böhm *et al.*, 2016; Coskun *et al.*, 2016*b*), also did not change in response to Si provision in either cultivar (Fig. 4B).

**Fig. 5.**
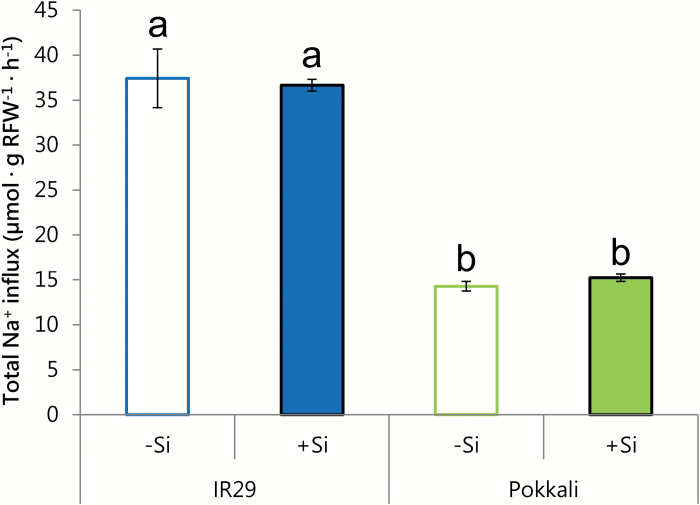
Unidirectional Na^+^ influx in 21-day-old seedlings of IR29 and Pokkali, grown and measured at 25 mM [Na^+^]_ext_ and 0 or 3 mM Si. Plants were loaded for 2 min in radioactive solution, then desorbed first for 5 s and then for 7 min in non-radioactive solution. Different lower case letters (a, b) indicate significant differences between conditions (*t*-test, *P*<0.05). Data are represented as the mean ±SEM (*n*=3). Measurements taken at 30 °C in seedlings grown under a 30/20 °C (day/night) temperature cycle. Unidirectional influx values, derived using compartmental analysis at cultivar-specific EC_50_s, can be found in Supplementary Fig. S4.

**Fig. 6.**
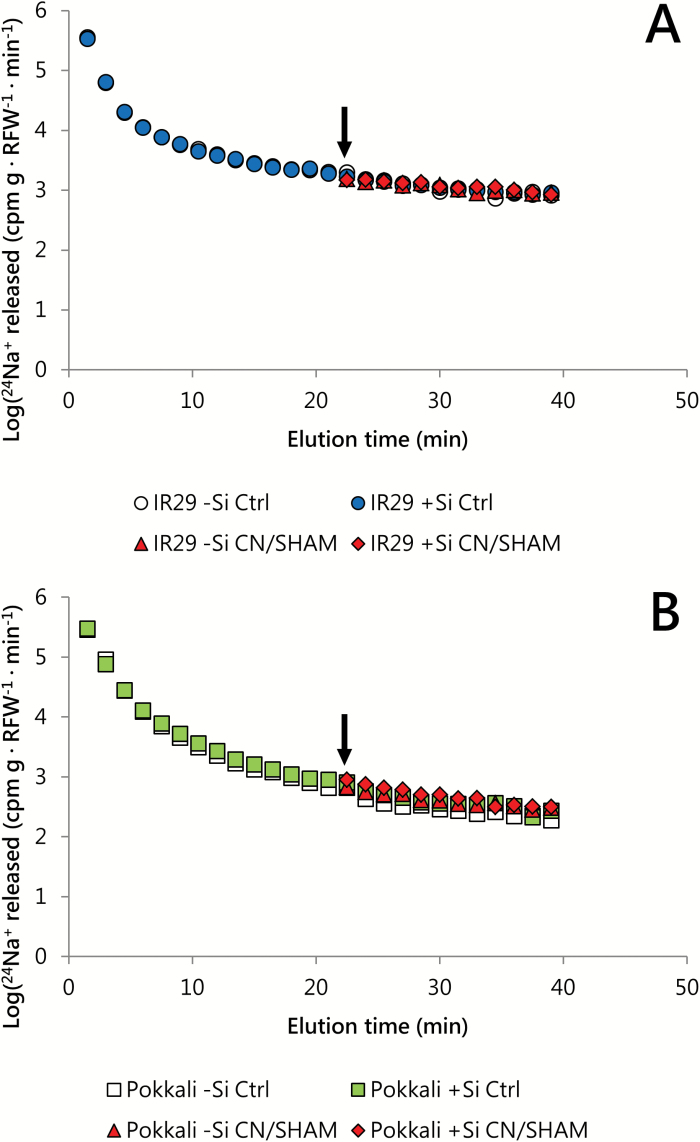
^24^Na^+^ efflux from roots of intact, 21-day-old seedlings of IR29 (A) and Pokkali (B), grown and measured at 35 mM and 50 mM [Na^+^]_ext_, respectively (cultivar Na^+^ EC_50_ values), with or without Si (1.67 mM). Respiratory inhibitors KCN and SHAM (when used) were applied at *t*=22.5 min (from the beginning of the elution protocol; see arrows) onwards. Data are represented as the mean (SEM<15% of the mean; *n*=3–7). Efflux and influx values calculated from these data are shown in Supplementary Fig. S4. Measurements taken at 30 °C in seedlings grown under a 30/20 °C (day/night) temperature cycle.

This suggests that Si may exert no control over the Na^+^ transport systems located in the plasma membranes of root cells, a possibility that was consistent with our finding that Si had no effect on unidirectional ^24^Na^+^ fluxes, either into (Fig. 5; Supplementary Fig. S4) or out of (Fig. 6; Supplementary Fig. S4) roots of IR29 and Pokkali, at 25 mM [Na^+^]_ext_ (Fig. 5), and at their respective EC_50_ values (Fig. 6; Supplementary Fig. S4). In other words, neither of the two component fluxes responsible for root Na^+^ cycling (influx and efflux) was affected by Si. Importantly, however, radiotracer methods of this sort, while commonly used, are less direct than electrophysiological methods (Fig. 4B), and, under low-affinity conditions, may not necessarily delineate fluxes across cellular membranes (Coskun *et al.*, 2016*b*). We have previously put forward arguments that, on the contrary, the extremely high, cyclical radiotracer fluxes of Na^+^ under saline conditions may have a large, indeed predominant, extracellular (apoplastic) character that, in the RTSC model, has been mistakenly interpreted to be a symplastic phenomenon (Britto and Kronzucker, 2009, 2015).

One of several lines of evidence contradicting the RTSC model (see the Introduction) is the strong resistance to change found in the efflux component of the Na^+^ cycle, even in the presence of powerful inhibitors (see Britto and Kronzucker, 2015, for details). The absence of inhibitory effects contrasts starkly with the high degrees of malleability found in efflux components of other plant membrane transport systems, such as those of high-affinity ammonium (NH_4_^+^; Britto and Kronzucker, 2003) and potassium (Coskun *et al.*, 2010) transport, and low-affinity ammonia (NH_3_) transport (Coskun *et al.*, 2013). The recalcitrance of low-affinity Na^+^ efflux is confirmed in Fig. 6, which shows no change in ^24^Na^+^ release from labeled roots of either cultivar, in response to the application of the respiratory blockers CN^–^ and SHAM (positive controls for these inhibitors, confirming their potency and rapidity of action, can be found in Supplementary Figs S5–S7; also see Britto and Kronzucker, 2015). The lack of response is difficult to explain in terms of the RTSC model, especially given that Na^+^ efflux is considered to be the thermodynamically active component of the ostensible membrane transport cycle (Munns and Tester, 2008). Our observations of Na^+^-dependent membrane depolarization (Fig. 4) do suggest that there is nevertheless at least a small, Si-insensitive, transmembrane flux of Na^+^ into root cells, which might be masked in radiotracer experiments by the much larger fluxes of a rapid, apoplastic, Na^+^ cycle. The magnitude of such a membrane flux may be of the order of 5–10 μmol g^−1^ (FW) h^−1^, given that the maximal depolarization observed here (65–75 mV) with low-affinity Na^+^ transport is quantitatively similar to that seen with low-affinity K^+^ transport (66 mV), for which such flux values have been obtained (Coskun *et al.*, 2016*b*).

If the rapid cycling of Na^+^ in roots is indeed an apoplastic phenomenon, it is of special interest that it is not affected by Si, given that Si can so strongly suppress transpirational bypass flow, which is also thought to be an apoplastic phenomenon in many or most cultivars of rice (Yeo *et al.*, 1999; Faiyue *et al.*, 2012). The lack of an Si effect on putative cycling in the root apoplast may be due to the preferential accumulation of Si around the endodermis in roots (Parry and Soni, 1972; Lux *et al.*, 1999; Gong *et al.*, 2006), whereas the bulk of apoplastic Na^+^ cycling is likely to occur in the cortex. Si is also known to accumulate in the exodermis (Gong *et al.*, 2006; cf. Lux *et al.*, 1999) but, due to the relatively short lifespan of the plants used here (3 weeks), and to the fact that exodermal development is reduced under hydroponics (Krishnamurthy *et al.*, 2009; Meyer *et al.*, 2009), exodermal Si barriers would probably not have been well enough developed, in the present study, to limit Na^+^ flow into and out of the cortex. In the presence of a well-developed exodermis, Si may indeed be found to exert an effect on Na^+^ cycling, and the role of plasma membrane transporters for Na^+^ in root cells might also be more important, at least under conditions where there is a plant sodium demand (e.g. when K^+^ availability is low). Further study will be required to assess these possibilities.

While some studies have shown that Si reduces the uptake of cadmium, arsenic, and phosphorus ions, it remains unclear as to whether this is due to competitive effects at the transporter level, or to alterations in the apoplast leading to extracellular ion binding (Guo *et al.*, 2007; Fleck *et al.*, 2013; Shi *et al.*, 2013; Ma *et al.*, 2015; Greger *et al.*, 2016). In our study, regardless of whether the phenomenon of rapid Na^+^ cycling in roots is interpreted as a symplastic or an apoplastic phenomenon, our tracer experiments do not support either competitive or extracellular binding effects of Si.

### Conclusion

**Fig. 7.**
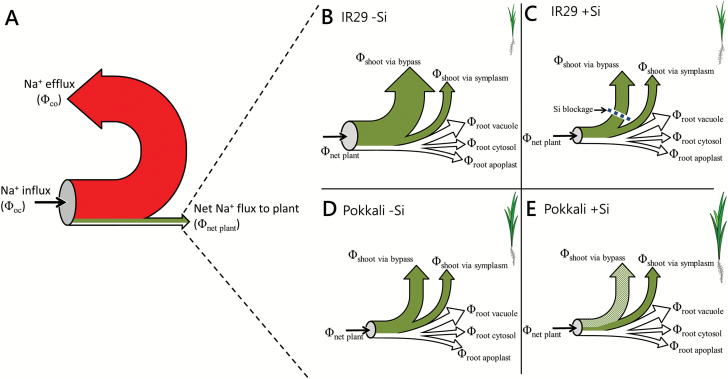
Model outlining Na^+^ transport scenarios in IR29 and Pokkali rice, under saline conditions, and the role of Si. The large magnitudes of unidirectional Na^+^ influx (ϕ_oc_) and efflux (ϕ_co_) depicted in (A) are responsible for root Na^+^ cycling; we hypothesize that this cycling occurs mainly in the apoplast. The much smaller resultant net flux into the plant (ϕ_net plant_, defined as the difference between ϕ_oc_ and ϕ_co_) branches into still smaller fluxes leading to Na^+^ accumulation in root cells (cytosol and vacuole), the root apoplast, and in the shoot, where it arrives both through bypass flow and via the symplasm into the xylem and then via the transpiration stream to the shoot (B–E). In IR29, there is a large contribution of apoplastic bypass flow of Na^+^ to the shoot under control (–Si) conditions (B), but bypass flow and Na^+^ accumulation in the shoot are both significantly decreased by Si treatment (C). In Pokkali, in contrast, there is low apoplastic bypass regardless of Si condition (D, E). In this cultivar, Si decreases shoot [Na^+^] by virtue of its stimulation of shoot growth, and the resulting dilution of incoming Na^+^ (represented by checkered fill of ϕ_shoot via bypass_, the flux to the shoot via apoplastic bypass; E). ϕ_shoot via symplasm_, flux via the symplasm to the xylem and then to the shoot; ϕ_root vacuole_, flux to the root vacuole; ϕ_root cytosol_, flux to the root cytosol; ϕ_root apoplast_, flux to the root apoplast.

Silicon has been long classified as a non-essential element in plant nutrition, and while routinely left out of nutrient solutions, it has nevertheless been shown to be substantially beneficial to a wide range of plants (Epstein, 1999; Coskun *et al.*, 2016*a*). However, the mechanisms by which these benefits are achieved are only just beginning to be unraveled. Clearly, in rice, which is a strong Si accumulator (Ma and Takahashi, 2002), Si can significantly improve growth under both benign and saline conditions. In both IR29 and Pokkali, we found Si to reduce Na^+^ levels strongly in leaves, but distinct mechanisms underlying this reduction appear to be operating in the two cultivars: blocking of bypass flow in the former, and growth dilution of Na^+^ in the latter (summarized in Fig. 7). In addition, other Si-triggered processes are likely to underlie improved growth of the two cultivars, possibly through changes in gene expression (Debona *et al.*, 2017). However, our study casts doubt on any influence Si may have on either Na^+^ transport across cell membranes or the rapid cycling of Na^+^ into and out of roots, whether by apoplastic or symplastic means. More broadly, much more work will be required to understand the beneficial effects of Si on plants, and to use this understanding to optimize the productivity of crucial crop species such as rice. In addition, much more remains to be discovered about Na^+^ dynamics across membranes and through extracellular volume in plants; indeed, many questions must still be asked about the magnitudes and underlying mechanisms of the various Na^+^ fluxes summarized in Fig. 7, and their relationships to Na^+^ stress and toxicity.

## Supplementary data

Supplementary data are available at *JXB* online.

Fig. S1. Dry weights of 21-day-old IR29 and Pokkali seedlings grown at 5–50 mM NaCl and with or without Si (1.67 mM).

Fig. S2. Dry weights and water content in shoot, root, and whole plant of 21-day-old IR29 and Pokkali seedlings grown at 5–50 mM NaCl and with or without Si (1.67 mM).

Fig. S3. Dry:fresh weight ratios of shoot, root, and total biomass in 21-day-old IR29 and Pokkali seedlings grown at 5–50 mM NaCl and with or without Si (1.67 mM).

Fig. S4. Unidirectional influx and efflux of Na^+^ (as measured with compartmental analysis by tracer efflux) in 21-day-old IR29 and Pokkali seedlings grown and measured at 35 mM and 50 mM NaCl (respective cultivar-specific Na^+^ EC_50_ values) with or without Si (1.67 mM).

Fig. S5. Effect of NaCN and SHAM on net K^+^ fluxes in 21-day-old IR29 and Pokkali seedlings grown at 35 mM and 50 mM NaCl (respective cultivar-specific Na^+^ EC_50_ values) with or without Si (1.67 mM).

Fig. S6. Effect of KCN and SHAM (1 mM each) on root O_2_ consumption of 21-day-old Pokkali seedlings grown at 75 mM NaCl in the absence of Si.

Fig. S7. Electrical potential differences (∆Ψ) across the plasma membrane of root cells in 21-day-old seedlings of IR29 and Pokkali rice grown with or without Si (1.67 mM) and as affected by KCN and SHAM (1 mM each), under either non-saline conditions (5 mM) or at 35 mM and 50 mM NaCl (respective cultivar-specific Na^+^ EC_50_ values).

Table S1. Two-way ANOVA examining the effect of Si (1.67 mM), cultivar (IR29 and Pokkali), and their interaction on fresh weight of 21-day-old seedlings grown at 5–50 mM NaCl.

Table S2. Student’s *t*-test examining the effect of Si (1.67 mM) on fresh weight, dry weight, and water content of 21-day-old IR29 and Pokkali seedlings grown at 5–50 mM NaCl.

Table S3. Two-way ANOVA examining the effect of NaCl (5–50 mM), Si (1.67 mM), and their interaction on 21-day-old IR29 and Pokkali seedling fresh weight and ion concentration.

Table S4. Student’s *t*-test examining the effect of Si (1.67 mM) on shoot, root, and whole-plant Na^+^:K^+^ ratios in 21-day-old IR29 and Pokkali seedlings grown at 5–50 mM NaCl.

Table S5. Values for transpirational bypass flow in rice from the present work and previously published studies.

Supplementary Tables Figures

## Abbreviations

CATE: compartmental analysis by tracer efflux
EC_50_: half maximal effective concentration
[Na^+^]_ext_: external sodium concentration
PTS: trisodium 8-hydroxypyrene-1,3,6-trisulfonic acid
RTSC: rapid trans-membrane sodium cycling
SHAM: salicylhydroxamic acid
