# Supplementary material for: Membrane fluxes, bypass flows, and sodium stress in rice: the influence of silicon
Source: J Exp Bot. 2018 Jan 11;69(7):1679–92. doi: 10.1093/jxb/erx460 (PMC5889039; doi:10.1093/jxb/erx460)
Supplement: Supplementary Tables Figures [file erx460_suppl_supplementary_tables_figures.pdf]

Table S1. Effect of Si, cultivar, and their interaction on plant fresh weight at different $[Na^+]_{ext}$ levels examined using two-way ANOVA.

	$[Na^+]_{ext}$	[Si]	Cultivar	[Si] + Cultivar Interaction
Shoot FW[1]	5	****	****	-
	10	*	****	-
	25	**	****	-
	50	****	****	-
Root FW	5	-	-	-
	10	-	-	-
	25	-	-	-
	50	***	****	-
Total FW	5	**	****	-
	10	-	****	-
	25	-	****	-
	50	****	****	-

Si provided at 0 or 1.67 mM, $[Na^+]_{ext}$ (as NaCl) from 5-50 mM. Cultivars compared were IR29 and Pokkali. Asterisks denote different levels of significance (-: no significance, *: $P < 0.05$, **: $P < 0.01$, ***: $P < 0.005$, ****: $P < 0.001$).

[1]fresh weight

Table S2. Effect of Si on total fresh weight, dry weight, and water content at different $[Na^+]_{ext}$ levels examined using Student's t-test with Bonferroni *post hoc*.

$[Na^+]_{ext}$	Cultivar	-/+ Si	FW[1] (g per plant)				DW[2] (g per plant)				WC[3] (%)			
5 mM	IR29	-Si	0.579	±	0.042		0.0813	±	0.006	***	86.0	±	0.1	****
		+Si	0.680	±	0.060		0.1085	±	0.009	***	83.9	±	0.2	****
	Pokkali	-Si	0.759	±	0.051	**	0.0962	±	0.010	****	87.2	±	1.0	***
		+Si	0.965	±	0.064	**	0.1514	±	0.009	****	84.1	±	0.5	***
10 mM	IR29	-Si	0.572	±	0.039		0.0827	±	0.005	***	85.4	±	0.2	****
		+Si	0.673	±	0.060		0.1110	±	0.010	***	83.5	±	0.1	****
	Pokkali	-Si	0.993	±	0.068		0.1401	±	0.008	***	85.7	±	0.3	****
		+Si	1.096	±	0.086		0.1775	±	0.011	***	83.5	±	0.6	****
25 mM	IR29	-Si	0.418	±	0.019		0.0616	±	0.003	*	85.3	±	0.2	****
		+Si	0.443	±	0.037		0.0754	±	0.006	*	83.0	±	0.2	****
	Pokkali	-Si	0.705	±	0.045		0.1011	±	0.005	***	85.5	±	0.2	***
		+Si	0.846	±	0.064		0.1413	±	0.010	***	83.0	±	0.6	***
50 mM	IR29	-Si	0.140	±	0.016	***	0.0261	±	0.002	***	80.5	±	0.7	
		+Si	0.219	±	0.021	***	0.0407	±	0.004	***	81.2	±	0.3	
	Pokkali	-Si	0.421	±	0.020	***	0.0653	±	0.003	****	84.4	±	0.3	***
		+Si	0.655	±	0.072	***	0.1097	±	0.012	****	83.2	±	0.1	***

Si provided at 0 or 1.67 mM, $[Na^+]_{ext}$ (as NaCl) from 5-50 mM. Asterisks denote different levels of significance for comparisons between control (-Si) and +Si conditions within a $[Na^+]_{ext}$ level and cultivar (*: $P < 0.05$, **: $P < 0.01$, ***: $P < 0.005$, ****: $P < 0.001$). Water content was calculated as (FW-DW)/DW×100.

[1]fresh weight; [2]dry weight; [3]water content

Table S3. Effect of $[Na^+]_{ext}$, Si, and their interaction on plant fresh weight and ion concentrations examined using two-way ANOVA.

		$[Na^+]_{ext}$	[Si]	[Na] + [Si] interaction
IR29	Shoot FW[1]	****	***	-
	Root FW	****	-	-
	Total FW	****	**	-
	Shoot $[Na^+]$	****	****	****
	Root $[Na^+]$	****	-	-
	Shoot $[K^+]$	****	-	-
	Root $[K^+]$	****	-	-
Pokkali	Shoot FW	****	****	-
	Root FW	****	-	-
	Total FW	****	****	-
	Shoot $[Na^+]$	****	****	***
	Root $[Na^+]$	****	-	-
	Shoot $[K^+]$	****	-	-
	Root $[K^+]$	****	-	-

Si provided at 0 or 1.67 mM, $[Na^+]_{ext}$ (as NaCl) from 5-50 mM. Asterisks denote different levels of significance (-: no significance, *: $P < 0.05$, **: $P < 0.01$, ***: $P < 0.005$, ****: $P < 0.001$).

[1]fresh weight

Table S4. Effect of Si on shoot Na^+:K^+, root Na^+:K^+, and total Na^+:K^+ at different $[Na^+]_{ext}$ levels examined using Student's t-test with Bonferroni *post hoc*.

		IR29		Pokkali	
	$[Na^+]_{ext}$	-Si	+Si	-Si	+Si
Shoot Na^+:K^+	**5**	0.061*	0.03*	0.066	0.039
	**10**	0.126**	0.061**	0.064	0.044
	**25**	0.599****	0.243****	0.242****	0.101****
	**50**	2.866**	1.393**	0.751***	0.363***
Root Na^+:K^+	**5**	0.179	0.193	0.257	0.276
	**10**	0.368	0.344	0.454	0.517
	**25**	1.104	1.241	1.042	0.975
	**50**	2.536	2.663	1.625	1.831
Total Na^+:K^+	**5**	0.105	0.095	0.144	0.136
	**10**	0.220**	0.162**	0.212	0.216
	**25**	0.803***	0.501***	0.520*	0.359*
	**50**	2.832**	1.637**	0.992***	0.723***

Si provided at 0 or 1.67 mM, $[Na^+]_{ext}$ (as NaCl) ranged from 5 to 50 mM. Asterisks denote different levels of significance for comparisons between control (-Si) and +Si conditions within a $[Na^+]_{ext}$ level and cultivar (*: $P < 0.05$, **: $P < 0.01$, ***: $P < 0.005$, ****: $P < 0.001$).

Table S5. Values for transpirational bypass flow in rice, from present work and previously published studies.

Study	Cultivar	Bypass flow (% of transpiration)
Flam-Shepherd *et al.* (present study)	IR29 (-Si)	5.6
	IR29 (+Si)	1.02
	Pokkali (-Si)	1.5
	Pokkali (+Si)	1.17
Gong *et al.* 2006	IR36 (-Si)	4.2
	IR36 (+Si)	0.8
Garcia *et al.* 1997	IR36	5.47
Yeo *et al.* 1987	IR26, IR2153-26-3-5-2	0.5-1.0*
Faiyue *et al.* 2010b	IR36	1.33-2.91**
Faiyue *et al.* 2012	IR55178 (low-Na^+-transporting lines)	1.37
	IR55178 (low-Na^+-transporting lines)	3.29
Faiyue *et al.* 2010a	Oochikara	0.86
	Nipponbare	1.61
	Taichung 65	0.7
Anil *et al.* 2005	Pokkali (low Na^+, high Ca^{2+})	8.87***
	Pokkali (high Na^+, low Ca^{2+})	54.1***
	Jaya (low Na^+, high Ca^{2+})	21.39***
	Jaya (hig Na^+, low Ca^{2+})	68.25***

* median values

**measured between 30 and 90% RH (bypass flow was inversely proportional to RH)

***The values in this study are up to two orders of magnitude larger than found in other work, possibly due to the extremely low transpiration values reported (less than 1 mL H_2O transpired per day by a block of 7 2-week-old plants).

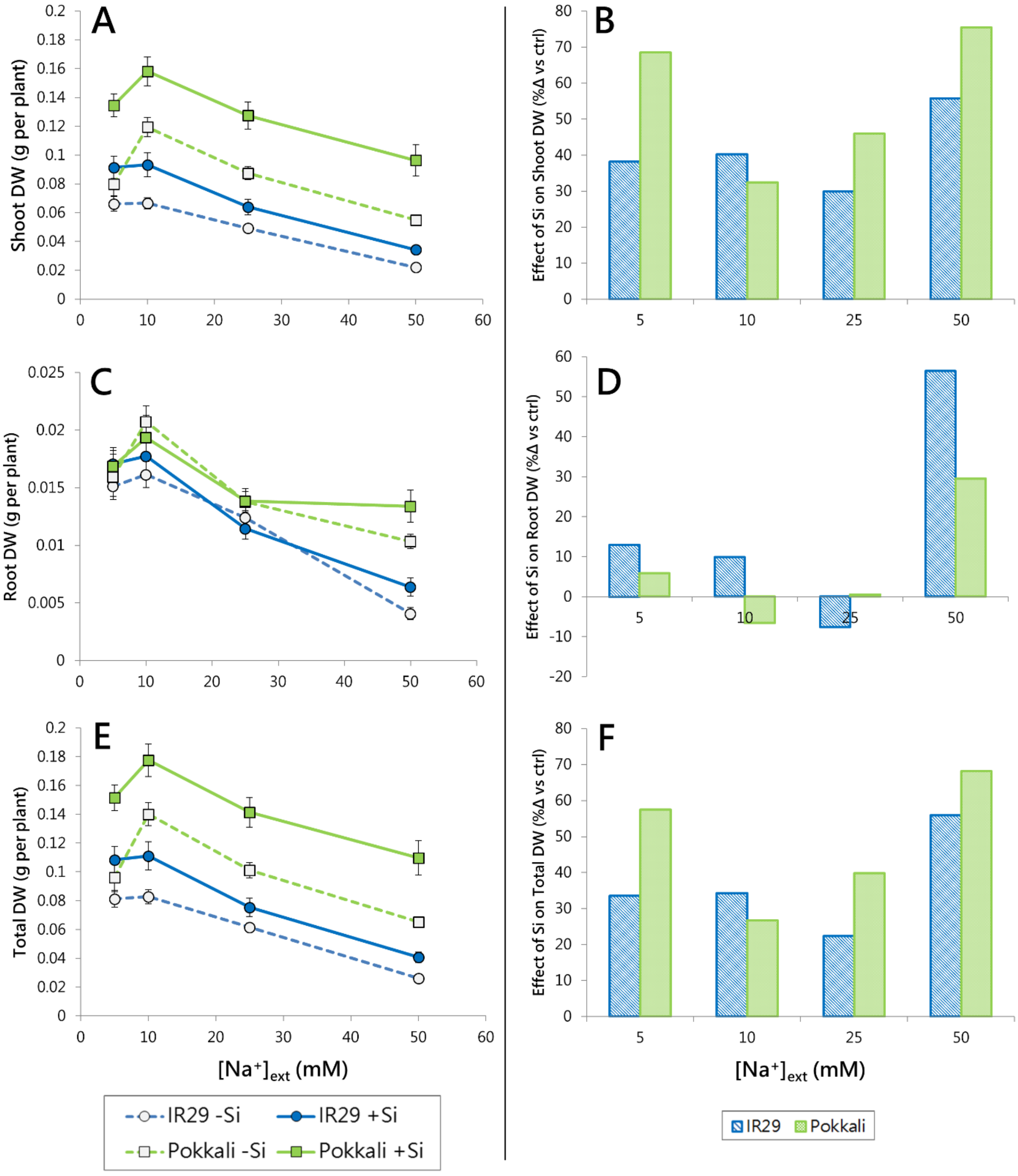

A
B
C
D
E
F
Shoot DW (g per plant)
Root DW (g per plant)
Total DW (g per plant)
Effect of Si on Shoot DW (%Δ vs ctrl)
Effect of Si on Root DW (%Δ vs ctrl)
Effect of Si on Total DW (%Δ vs ctrl)
[Na+]ext (mM)
IR29 -Si
IR29 +Si
Pokkali -Si
Pokkali +Si
IR29
Pokkali

Fig. S1. Effects of $[Na^+]_{ext}$ (5-50 mM; as NaCl) and Si (0 or 1.67 mM) on IR29 (circular symbols, blue lines) and Pokkali (square symbols, green lines) shoot (A), root (C), and total DW (E), and the ratio of Si-treated DW to control DW of shoot (B), root (D), and total plant DW (F), expressed as percentages. DW data are represented as mean ± SEM (n = 11-12). Control conditions (-Si) are represented using dotted lines and empty symbols, while +Si conditions are represented using solid lines and filled-in symbols, where the colour of the lines and the symbols (where applicable) correspond to the cultivar-specific colours described above. Measurements were collected during the day from 21-day-old seedlings grow at 30 ^oC and 20 ^oC in the daytime and nighttime, respectively.

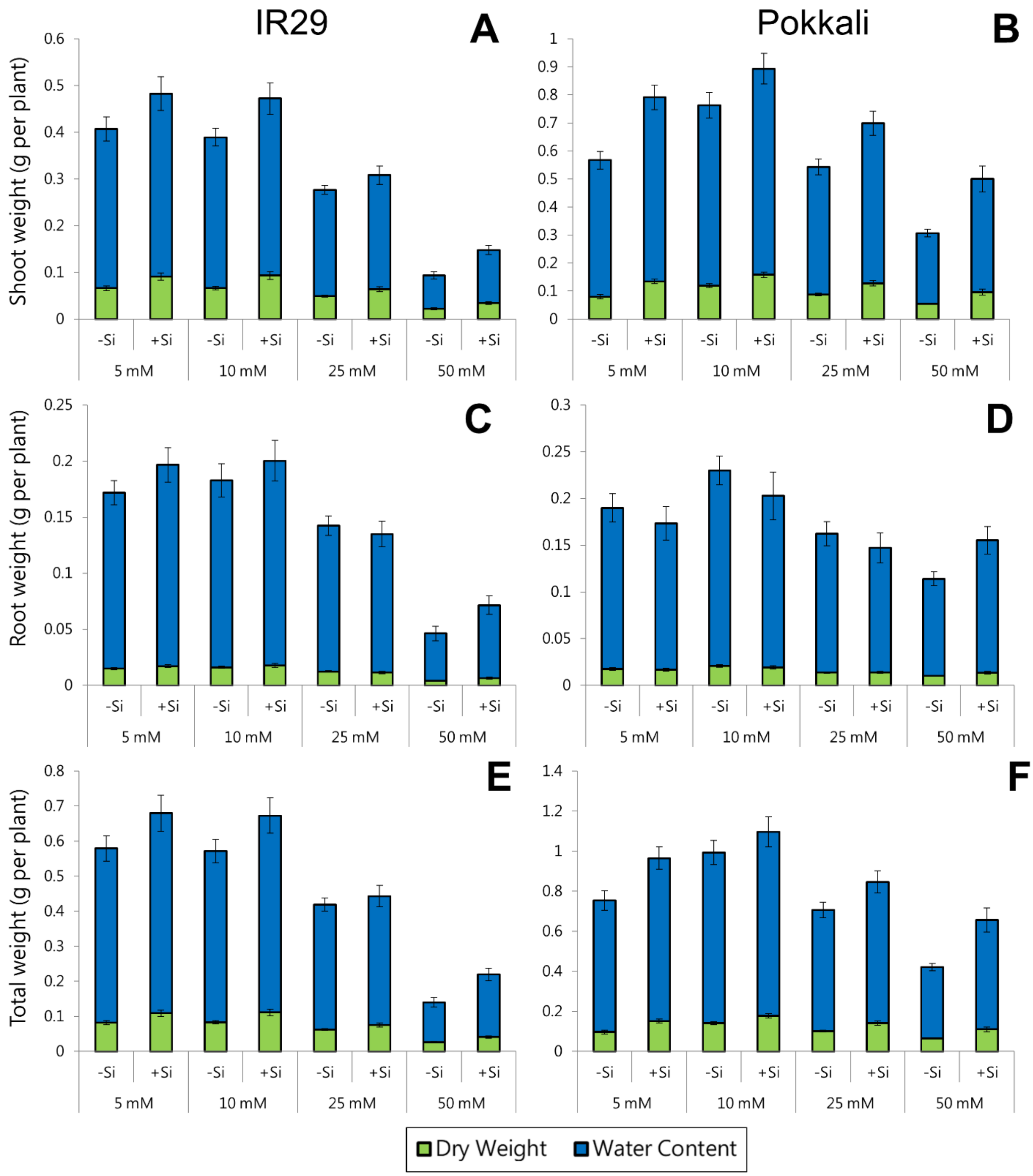

IR29
A
Pokkali
B
C
D
E
F
Shoot weight (g per plant)
Root weight (g per plant)
Total weight (g per plant)
-Si
+Si
5 mM
10 mM
25 mM
50 mM
Dry Weight
Water Content

Fig. S2. Effects of $[Na^+]_{ext}$ (5-50 mM) and Si (0 or 1.67 mM) on dry weight (green) and water content (blue) of shoots (A and B), roots (C and D), and whole plants (E and F) of IR29 and Pokkali. Data are represented as mean ± SEM (n = 11-12). Measurements were collected during the day from 21-day-old seedlings grown at 30 ^{o}C and 20 ^{o}C in the daytime and nighttime, respectively.

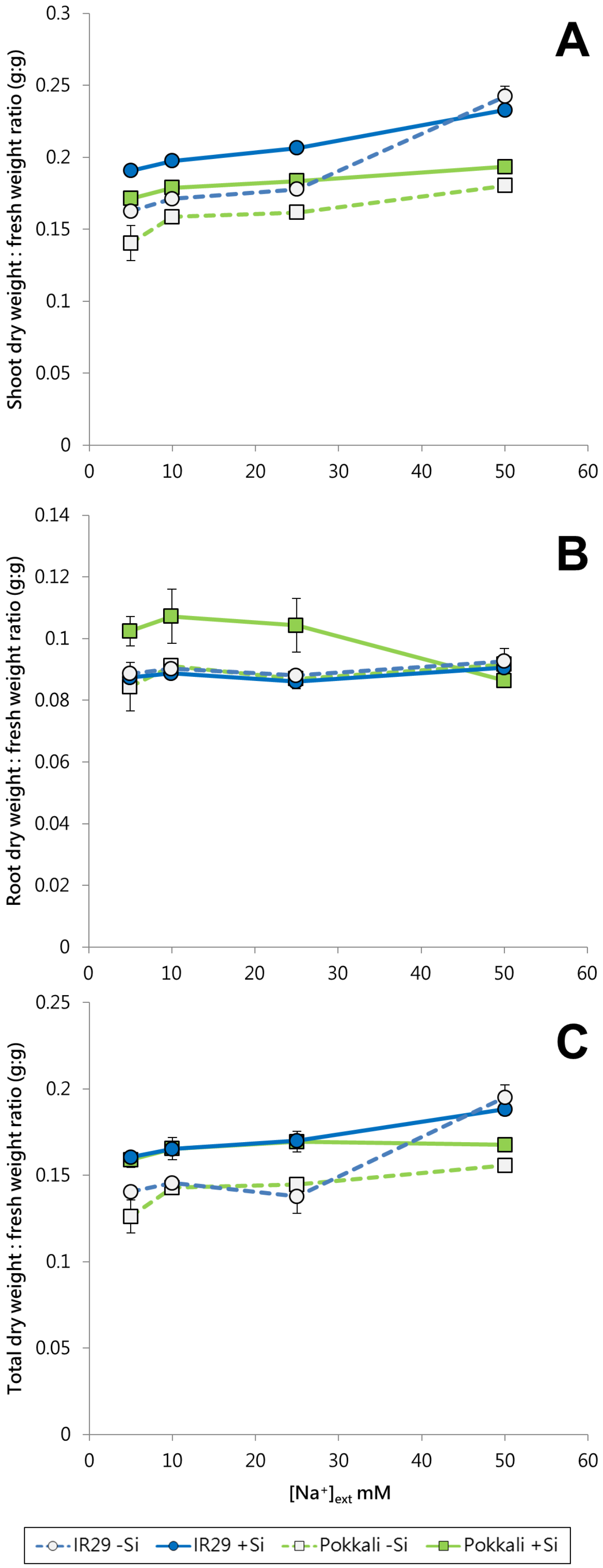

A
Shoot dry weight : fresh weight ratio (g:g)
0.3
0.25
0.2
0.15
0.1
0.05
0
0 10 20 30 40 50 60
B
Root dry weight : fresh weight ratio (g:g)
0.14
0.12
0.1
0.08
0.06
0.04
0.02
0
0 10 20 30 40 50 60
C
Total dry weight : fresh weight ratio (g:g)
0.25
0.2
0.15
0.1
0.05
0
0 10 20 30 40 50 60
[Na⁺]ext mM
IR29 -Si
IR29 +Si
Pokkali -Si
Pokkali +Si

Fig. S3. Effects of $[Na^+]_{ext}$ (5-50 mM) and Si (0 or 1.67 mM) on the shoot (A), root (B), and total (C) dry weigh:fresh weight ratios of IR29 and Pokkali. Data are represented as mean ± SEM (n = 11-12). Pokkali is represented using square symbols and green lines, while IR29 is represented using circular symbols and blue lines. Control conditions (-Si) are represented using dotted lines and empty symbols, while +Si conditions are represented using solid lines and filled-in symbols, where the colour of the lines and the symbols (where applicable) correspond to the cultivar-specific colours described above. Measurements were collected during the day from 21-day-old seedlings grow at 30 ^oC and 20 ^oC in the daytime and nighttime, respectively.

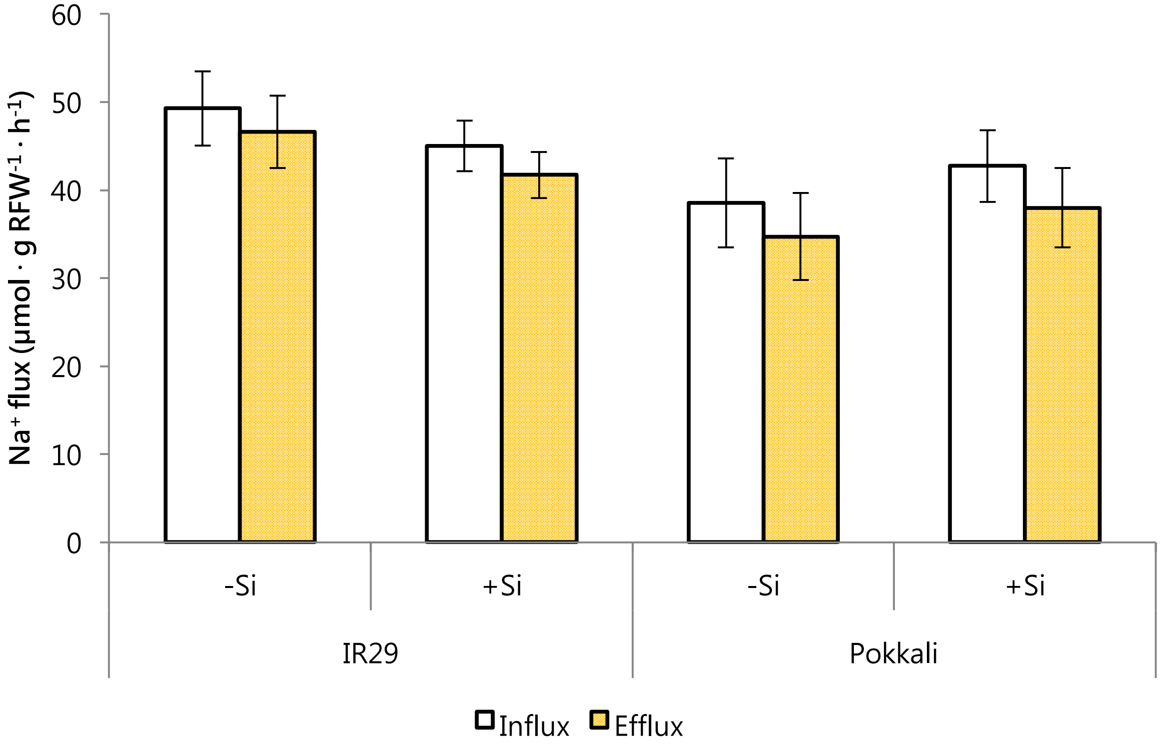

Fig. S4. Unidirectional influx and efflux of Na^+ in IR29 and Pokkali seedlings, grown and measured at 35 and 50 mM NaCl (respective cultivar-specific Na^+ EC_{50} values), with or without Si (1.67 mM). Measurements were made using compartmental analysis by tracer efflux. For each cultivar and unidirectional flux, no significant difference was seen when comparing +Si and -Si conditions. Data are represented as mean ± SEM (n = 5-7). Measurements were collected during the day from 21-day-old seedlings grow at 30 ^oC and 20 ^oC in the daytime and nighttime, respectively.

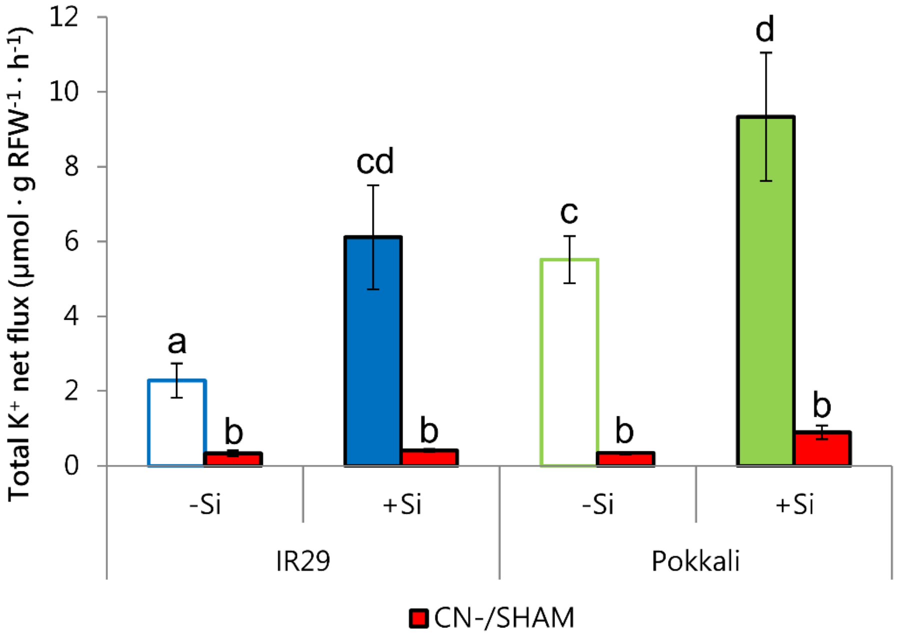

Fig. S5 Effects of Si (0 or 1.67 mM) and 1 mM NaCN and SHAM on total net K^+ flux in IR29 and Pokkali, grown at 35 and 50 mM $[Na^+]_{ext}$, respectively (cultivar Na^+ EC_{50}). Plants were loaded for 1 h in radioactive solution, either in control conditions or in the presence of CN^-/SHAM. Different lowercase letters (a, b) indicate significant differences between conditions (t-test, $P < 0.05$). Measurements were collected during the day from 21-day-old seedlings grown at 30 ^oC and 20 ^oC in the daytime and nighttime, respectively. Data are represented as mean ± SEM (n = 3).

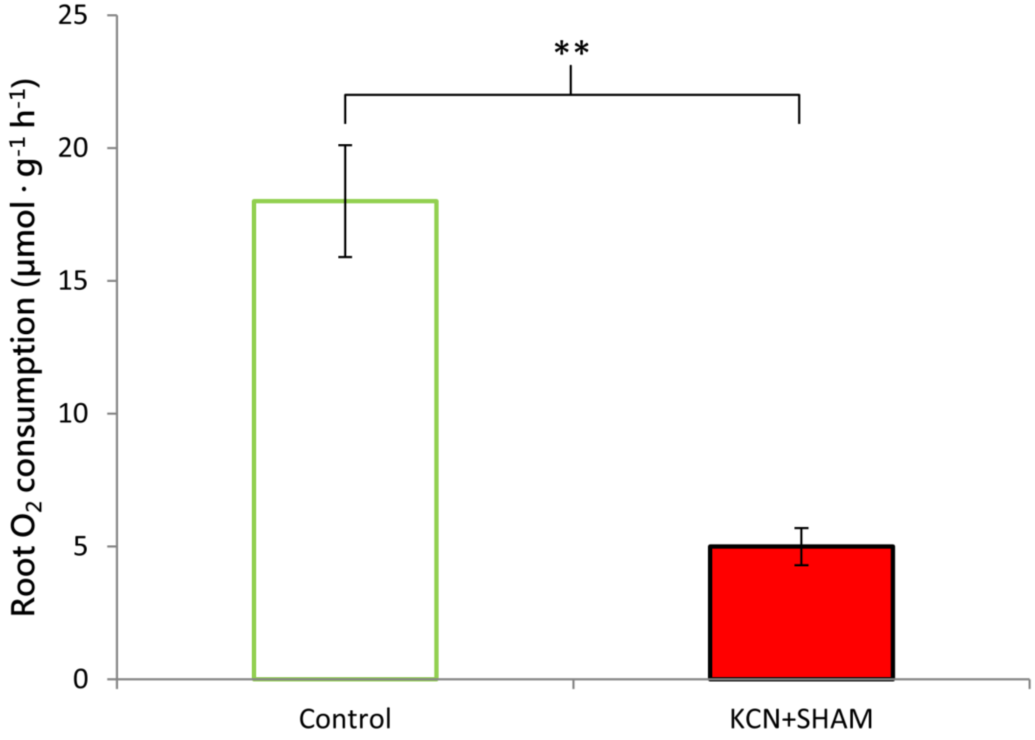

Fig. S6. Effects of KCN and SHAM on root O_2 consumption of Pokkali, grown at 75 mM $[Na^+]_{ext}$ in the absence of Si. Asterisks indicate significant differences between conditions (t-test; *: $P < 0.05$, **: $P < 0.01$). Data are represented as mean ± SEM (n = 5-10). Measurements were collected during the day from 21-day-old seedlings grow at 30 ^oC and 20 ^oC in the daytime and nighttime, respectively.

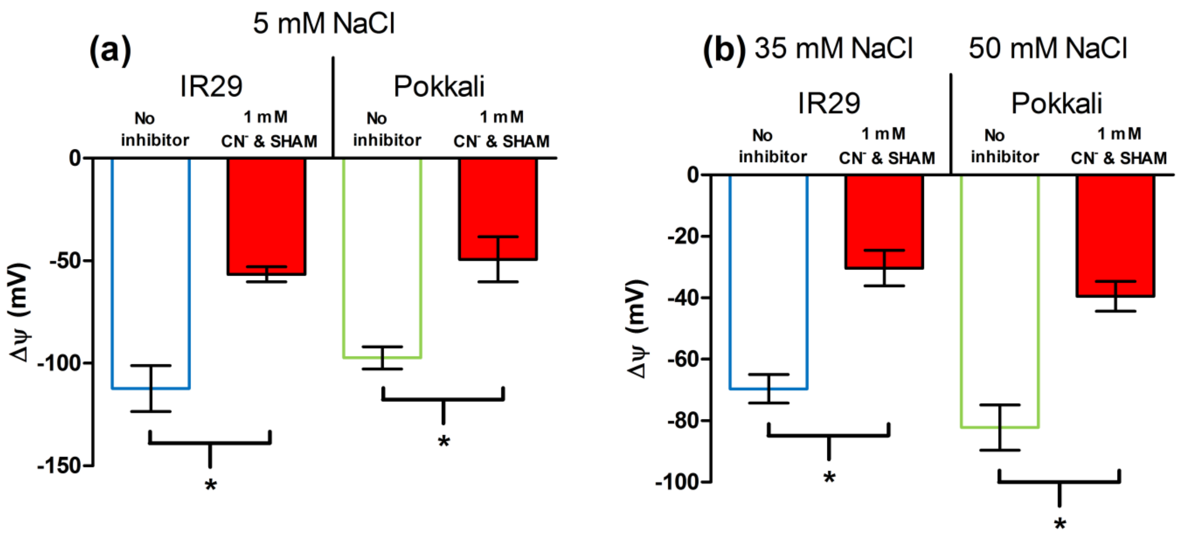

Fig. S7. Effects of Si (0 or 1.67 mM) and 1 mM KCN and SHAM on membrane potential differences at 5 mM $[Na^+]_{ext}$ (A), or at 35 and 50 mM $[Na^+]_{ext}$ for IR29 and Pokkali, respectively (cultivar Na^+ EC_{50}) (B). Data are represented as mean ± SEM (n = 3-4). Asterisks indicate significant differences between conditions (t-test; $P < 0.05$). Measurements were collected during the day from 21-day-old seedlings grow at 30 ^oC and 20 ^oC in the daytime and nighttime, respectively.
